# Guggulsterone Mediated JAK/STAT and PPAR-Gamma Modulation Prevents Neurobehavioral and Neurochemical Abnormalities in Propionic Acid-Induced Experimental Model of Autism

**DOI:** 10.3390/molecules27030889

**Published:** 2022-01-28

**Authors:** Rishabh Khera, Sidharth Mehan, Sonalika Bhalla, Sumit Kumar, Abdulrahman Alshammari, Metab Alharbi, Satya Sai Sadhu

**Affiliations:** 1 Department of Pharmacology, Neuropharmacology Division, ISF College of Pharmacy, Moga 142001, Punjab, India; rishabhkhera61199@gmail.com (R.K.); Sonalikabhalla97@gmail.com (S.B.); sumitsrivastav223@gmail.com (S.K.); 2 Department of Pharmacology and Toxicology, College of Pharmacy, King Saud University, P.O. Box 2455, Riyadh 11451, Saudi Arabia; abdalshammari@ksu.edu.sa (A.A.); mesalharbi@ksu.edu.sa (M.A.); 3 Chemistry Department, Northern Michigan University, 1401, Presque, Isle, Marquette, MI 49855, USA; ssadhu@nmu.edu

**Keywords:** autism, JAK/STAT, PPAR-gamma, guggulsterone, propionic acid, neuroprotection

## Abstract

Autism spectrum disorder is a neurodevelopmental disorder marked by repetitive behaviour, challenges in verbal and non-verbal communication, poor socio-emotional health, and cognitive impairment. An increased level of signal transducer and activator of transcription 3 (STAT3) and a decreased level of peroxisome proliferator-activated receptor (PPAR) gamma have been linked to autism pathogenesis. Guggulsterone (GST) has a neuroprotective effect on autistic conditions by modulating these signalling pathways. Consequently, the primary objective of this study was to examine potential neuroprotective properties of GST by modulating JAK/STAT and PPAR-gamma levels in intracerebroventricular propionic acid (ICV PPA) induced experimental model of autism in adult rats. In this study, the first 11 days of ICV-PPA injections in rats resulted in autism-like behavioural, neurochemical, morphological, and histopathological changes. The above modifications were also observed in various biological samples, including brain homogenate, CSF, and blood plasma. GST was also observed to improve autism-like behavioural impairments in autistic rats treated with PPA, including locomotion, neuromuscular coordination, depression-like behaviour, spatial memory, cognition, and body weight. Prolonged GST treatment also restored neurochemical deficits in a dose-dependent manner. Chronic PPA administration increased STAT3 and decreased PPAR gamma in autistic rat brain, CSF, and blood plasma samples, which were reversed by GST. GST also restored the gross and histopathological alterations in PPA-treated rat brains. Our results indicate the neuroprotective effects of GST in preventing autism-related behavioural and neurochemical alterations.

## 1. Introduction

Autism Spectrum Disorder (ASD) is a developmental disorder characterized by persistent social difficulties and restricted and repetitive behavioural patterns [1]. ASD is a spectrum disorder because it manifests differently in each patient. These issues frequently appear in early childhood and tend to hinder functioning in various circumstances [2]. According to the Centers for Disease Control and Prevention, one out of every 54 children is affected by this increasingly widespread condition [3]. Early-onset autism symptoms begin immediately after birth, while regressive autism symptoms start around the age of two and then progress slows down [4].

JAK-STAT signalling primarily involves gene regulation, hormone release, inflammation, cell proliferation, and differentiation in the central nervous system [5]. Long-term memory necessitates cytokine signalling via the JAK/STAT pathway [6]. The JAK/STAT signalling system is essential in developing and regulating immune responses [7]. This pathway is also implicated in a variety of neurodegenerative diseases, including Parkinson’s disease [8], epilepsy [9], amyotrophic lateral sclerosis, Huntington’s disease [10], multiple sclerosis [11], and neuroinflammation [12]. The STAT3 inhibitor S3I-201 restored neural-immune function in BTBR animal models [13] and downregulated chemokine receptors, inflammatory biomarkers, and lymphocyte activation markers [14]. Additionally, diosmin and luteolin have been used as STAT3 inhibitors to treat autism [15]. Ahmad and his colleagues investigated AG490 as another possible treatment for autism [14]. JAK/STAT inhibitors are also used for the treatment of a variety of neurological diseases, including simvastatin for multiple sclerosis (MS) patients [16], SSRIs utilized for depression patients [17], pacritinib for Glioblastoma multiforme (GBM) clinically [18]. Moreover, AZD1480 was being used preclinically for Parkinson’s Disease (PD) [19] and Stattic for Alzheimer’s disease [20].

PPAR gamma is a nuclear receptor that regulates various physiological processes such as insulin sensitivity, adipogenesis, and inflammation [21]. PPAR gamma controls placental vascularization, myocardial development, and monocyte differentiation [22]. PPAR-gamma controls fatty acid synthesis, glucose metabolism, and adipocyte differentiation [23]. PPAR gamma activation may protect against neurotoxicity [24], neurodegeneration, drug dependency [25], and neuroinflammation [26]. It was also observed that PPAR gamma enhances antioxidant defence, mitochondrial biogenesis, and oxidative phosphorylation [27]. Rosiglitazone, a PPAR gamma agonist, has been shown to be effective in treating neuroinflammation [28], Alzheimer’s disease [29], and dyskinesia [30]. Pioglitazone has also been shown to have neuroprotective properties against autism [31,32].

Mifepristone, a PPAR gamma agonist, has also been shown to protect against cerebral ischemia [33]. PPAR gamma also activates the (SOCS) suppressor of cytokine signalling [34]. Increased SOCS 3 expression is associated with reduced IL-6 and TGF-1 expression, oedema formation, and vacuolization, most likely due to direct control on JAK2/STAT3 [35]. PPAR gamma agonists were reported to decrease inflammation in the brain by lowering the phosphorylation of JAK 1, 2, STAT 1, and 3 phosphorylation and rapidly promoting SOCS 1 and 3 in activated astrocytes and microglial cells [36]. An endogenous PPAR gamma ligand, 15d-PGJ2, has been shown to inhibit INF gamma induced chemokine protein synthesis and the JAK/STAT signalling pathway [37].

Guggulsterone (GST) is a component of guggulipid, an ethyl acetate extract of the Burseraceae plant *Commiphora whighitii*. In Ayurveda, it is used to treat a variety of conditions, including lipid disorders, obesity, and diseases such as rheumatoid arthritis [38]. GST has already been studied as an antidepressant [39], to diminish scopolamine-induced memory impairment [40], and to have anti-inflammatory effects in a range of hyperlipidemic situations [41].

GST was demonstrated to have neuroprotective potential and aid in restoring several behavioural and neurochemical abnormalities in an ethidium bromide-induced experimental model of multiple sclerosis in rats by Kumar and colleagues [42]. GST has been shown to be beneficial in treating a wide range of chronic diseases, including gastric cancer [43], diabetes [44], hyperlipidaemia [45], and nodulocystic acne [46].

Guggulsterone has anti-inflammatory, antioxidant, cardioprotective, neuroprotective, and anticancer properties [47]. GST, a farnesoid X receptor (FXR) antagonist, suppresses both inducible and constitutive STAT3 activation by activating Src homology-2 domain-containing protein tyrosine phosphatase 1 (SHP-1) [48]. The previous study has linked GST to a decrease in phosphorylated STAT3, which has been linked to a reduction in squamous cell carcinoma [49] and angiogenesis in colon cancer [50]. To summarise GST’s interaction with potential targets, Cornick and his colleagues confirmed GST’s connection with an increased level of PPAR gamma against diabetes in Lep(ob)/Lep(ob) mice, which leads to its activation [51]. Previous research on disorders such as obesity [52] and Graves’ orbitopathy [53] discovered that GST enhanced PPAR gamma mRNA and protein levels, therefore improving the disease condition.

As a result, our study will explore the neuroprotective potential of the JAK-STAT/PPAR gamma pathway modulator GST in a rat PPA-induced experimental model of autism. The efficacy of GST to treat autistic rats’ behavioural deficits and neurochemical alterations in brain homogenate, blood plasma, and CSF would be investigated. GST’s neuroprotective action would also be demonstrated by repairing morphological, gross pathological, and histological changes after PPA infusions.

## 2. Materials and Methods

### 2.1. Experimental Animals

Adult Wistar rats (250–300 g, six months old, total animal 36, total group six, each group containing six animals: 18 male and 18 female) were obtained from the Central Animal House, ISF College of Pharmacy, Moga, Punjab, India. All the animals were kept in standard conditions of 12 h reverse light-cycle with food and water (*ad libitum*) under room temperature at 23 ± 2 °C. They were housed in polyacrylic cages with wire mesh at the top and soft bedding to avoid any harm or injury. All the animals have acclimatized adequately to laboratory conditions before the experiment. The experimental protocol was approved by the Institutional Animal Ethics Committee (IAEC) with registration no. 816/PO/ReBiBt/S/04/CPCSEA as protocol no. ISFCP/IAEC/CPCSEA/Meeting No: 27/2020/Protocol No. 456 as per the guidelines of the Government of India.

### 2.2. Drugs and Chemicals

PPA was purchased from Sigma–Aldrich (St. Louis, MI, USA). GST was provided as an ex-gratia sample from BAPEX, New Delhi, India. All other chemicals used in the study were of analytical grade. Solutions of the drugs and chemicals were freshly prepared before use. GST was administered orally as an aqueous suspension with 1% carboxymethylcellulose [42].

### 2.3. Experimental Grouping of Animals

**Figure 1:**
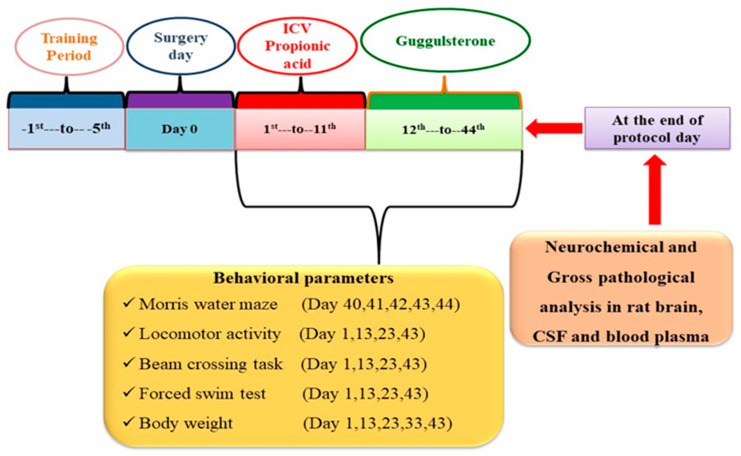
Experimental protocol schedule.

The total experimental protocol schedule was of 44 days [42]. Animals were divided into six groups. Group 1 Vehicle Control; Group 2 Sham control; Group 3 GST *perse* (60 mg/kg, p.o.); Group 4 PPA (10 µL/0.26 M, i.c.v.); Group 5 PPA (10 µL/0.26 M, i.c.v.) + GST (30 mg/kg, p.o.); Group 6 PPA (10 µL/0.26 M, i.c.v.) + GST (60 mg/kg, p.o.). The intra cerebral-ventricular injection (ICV) of PPA was given to induce autism. From day 1st to 11th, ICV-PPA was given, and then from day 12th until day 44th, drug GST was given orally at two doses (30 mg/kg and 60 mg/kg). To investigate autistic behaviour on particular days, several behaviour parameters such as the Morris water maze (MWM), locomotor activity, beam crossing task (BCT), and forced swim test (FST) were performed. On the 45th day, after completing the protocol schedule, all animals were sacrificed by decapitation. Their brains were isolated for various biochemical estimations and gross pathology to determine the neurochemical changes and demyelination in the brain. The experimental protocol is summarized in Figure 1.

**Note** **1.** *To complete the in-vivo and in-vitro analysis, all groups were not being treated at the same time. Each experimental group will have a 28-day gap before their initiation of protocol; To execute treatment schedules and behavioural parameters smoothly, not all experimental groups will be done simultaneously to resolve the experimental error*.

### 2.4. Propionic Acid-Induced Experimental Animal Model of Autism

The PPA-induced experimental model of autism in the rat was conducted according to the method established by [54]. The rat was first acclimatized to the laboratory conditions and then anesthetized by intraperitoneal ketamine injection at 75 mg/kg. The anesthetized rat’s body was then safely placed on a clean, warm padded surface. The head was allocated in a symmetrical position and secured with an incision bar and ear bars in the stereotaxic frame (Stoelting Co., Wood Dale, IL, USA). Before incision, the rat head was shaved, and fur was removed entirely from the scalp with 70% ethanol to avoid contamination. The scalpel then made a small incision (mid-sagittal), and the skull was exposed by removing all soft tissues. After skull exposure, the bregma and lambda were determined to facilitate the determination of the ICV injection coordinates. Cotton buds that were immersed in normal saline were used to prevent any bleeding and dehydration.

Under the aseptic conditions, a burr hole was made in the skull carefully, and a 2.5 cm long cannula was implanted at the following coordinates concerning bregma: anterior/posterior (AP)—1.3 mm; medial/lateral (ML)—1.8 mm; dorsal/ventral (DV)—3.0 mm in the skull. Immediately after that, the cannula was completely secured to the skull using dental cement, and the incision was sutured using a surgical suture and sterile surgical needle. A removable plastic ear pin was used to seal the cannula and removed before each ICV injection. All rats were given gentamycin (40 mg/kg) intraperitoneally after surgery to avoid sepsis, and lignocaine gel was administered to the sutured area to relieve pain. Neosporin powder was dusted on the skin daily for three days to prevent bacterial skin infections.

From day 1 to 11, PPA (10 μL of a 0.26 M solution) was injected through a 0.4 mm outer diameter hypodermic needle attached to a Hamilton microliter syringe (10-μL) in the left lateral cerebral ventricle for 10 min at 1 μL/min duration [55]. The Hamilton^®^ microneedle was placed for an extra 5 min after injection to promote drugs’ diffusivity in CSF and prevent backflow. Rats were kept separately in polyacrylic cages with warm fabric and husk during post-operative therapy, and extra attention was given until spontaneous movement was recovered. After anaesthesia, around 2–3 h [56], room temperature was held at 25 ± 3 °C. Milk and glucose water were given in a timely manner for 2–3 days to prevent any physical trauma and dehydration after surgery. All rats were observed daily for general body conditions such as body weight, dehydration, infection, and any other physical change. After seven days, rats were given a proper diet and water intake.

### 2.5. Parameters Assessed

Measurement of Weight Variations.

#### 2.5.1. Bodyweight Measurement

Bodyweight was observed on the experimental protocol schedule days 1, 13, 23, 33, and 43. It was used to monitor any physical change after surgery and PPA administration on body weight [57].

#### 2.5.2. Measurement of Relative-Brain Body Weight

The relative brain-body weight ratio was determined on the 45th day of the experimental protocol schedule. The freshly extracted weight was divided by the animal’s total body weight on the 45th day just before sacrificing the animal [58].

### 2.6. Behavioural Parameters

#### 2.6.1. Beam Crossing Task (BCT)

Each animal’s motor coordination ability was tested by movement across a wooden beam on days 1, 13, 23, and 43 of the experimental protocol schedule. The spongy mat was placed below to protect the rat from any harm after falling. The number of slips in each trial was recorded, and additionally, the direction towards which the animal falls was observed against a cut-off time of five minutes [59].

#### 2.6.2. Locomotor Activity Assessment

Animals were tested for locomotor activity on days 1, 13, 23 and 43 using an actophotometer (INCO, Ambala, Haryana, India). The behaviour parameter was performed according to the method described by [60]. The animal was placed in a digital actophotometer equipped with photocells sensitive to infrared light. They are then observed in a square, the closed arena, for five minutes. A digital actophotometer value started as counts per 5 min [60].

#### 2.6.3. Forced Swim Test (FST)

FST was used to assess the depressed behaviour of rats on days 1, 13, 23, and 43 because depression is a predictor of suicidal thoughts or attempts in children with autism. The initial exposure of rats in the tank during the training phase is 15 min, and the second is 24 h later, with a 5-min exposure time. The testing period comprises a single six-minute exposure. The first two minutes function as a habituation phase, and the final four minutes being the test itself, which provides the length of immobility [61].

#### 2.6.4. Morris Water Maze Task (MWM)

The spatial learning and memory of animals were evaluated using the Morris water maze (INCO, Ambala) on days 40, 41, 42, 43, and 44 of the protocol schedule. Water filled a circular water tank to a depth of 40 cm, and the hidden platform was submerged down 2 cm in water. The temperature of the water was maintained at 25 ± 1 °C. During the training session, four trials per session were given for the five days from day 40th to 44th. The time taken by the rat to reach the hidden platform was observed within a maximum time of 120 s, which is also called Escape latency time. If the rat did not find the concealed platform within the allotted 120 s, it was gently placed on it and left there for 20 s. A probing test (day 44) was performed twenty-four hours following the acquisition phase by removing the platform. TSTQ (Time spent in target quadrant) was measured after rats were free to swim in the pool for 120 s. TSTQ represents the amount of memory consolidation that occurred after learning [62].

### 2.7. Neurochemical Estimations

Collection and preparation of biological samples.

#### 2.7.1. Brain Homogenate Preparation

Animals were decapitated on the 45th day of the treatment schedule, and their brains were extracted and cleaned in ice-cold isotonic saline solution. Brain samples were then homogenized with ten times (*w*/*v*) ice-cold 0.1 M phosphate buffer (7.4). The homogenate was centrifuged at 10,000× *g* for 15 min, the supernatant was separated, and aliquots were used for biochemical estimation [63].

#### 2.7.2. Blood Plasma Collection and Separation

Blood was taken from the orbital sinus on the 45th day of the treatment schedule before the animal was sacrificed. Chloroform was used to anaesthetize the rats. The animal was then scruffed with the non-dominant hand’s thumb and forefinger, and the skin surrounding the eye was pulled tight. A capillary was introduced in the eye’s medial canthus (30-degree angle to the nose). Blood was taken in Eppendorf tubes containing EDTA after piercing the plexus/sinus. The plasma was then separated by centrifugation at 10,000× *g* for 5 min at 4 °C [64].

#### 2.7.3. CSF Collection

Rats were profoundly sedated with sodium pentobarbital after blood collection. The rats’ heads were held in place using a holder to show the arachnoid membrane, and a skin incision was performed to expose the translucent dura mater. Directly inserting a 30-gauge needle at a 30° angle into the cisterna magna yielded a maximum volume of 100 µL CSF. The sample was centrifuged at 2000× *g* for 10 min at 4 °C within 20 min after being collected. The supernatant was kept at −80 °C after centrifugation until further examination [65].

### 2.8. Assessment of Molecular and Cellular Markers

#### 2.8.1. Estimation of STAT3 and PPAR-Gamma Level

STAT3 was assessed in a sample of rat’s brain homogenate [42] and CSF [66] according to the instructions provided by manufacturers of (E-EL-0118) ELISA assay Kits from Elabscience Biotechnology Inc, Wuhan, China. The level of PPAR-gamma was also quantified in brain homogenate [42] and CSF [67] using an (E-EL-R0724) ELISA kit from Elabscience Biotechnology Inc, Wuhan, China. The values expressed nM/µg protein in brain homogenate and ng/mL in CSF.

#### 2.8.2. Estimation of MBP (Myelin Basic Protein) Level

R-MBP was assessed in brain homogenate according to the manufacturer instructions provided by (EL-R0642/MBP; Elabsciences, Wuhan, Hubei, China) ELISA assay Kits. The values were expressed in µg/mg protein [68].

### 2.9. Assessment of Apoptotic Markers

#### Estimation of Caspase-3, Bax, and Bcl-2 Level

Caspase-3 was measured in brain homogenate [54] and blood plasma [69] along with Bax, which was also quantified in rat’s brain homogenate [63] and blood plasma [70] (E-EL-R0160/ Caspase-3; E-EL-R0098/Bax/Bcl2 Elabsciences, Wuhan, Hubei, China) by ELISA kit. According to instructions of the manufacturer of the assay kit (Elabsciences, China), the level of anti-apoptotic protein Bcl-2 was also computed in the sample of brain homogenate [57] and blood plasma [71]. The assay employs the enzyme immunoassay competitive method. The values are expressed in ng/gm protein in brain homogenate and ng/mL in blood plasma.

### 2.10. Assessment of Neurotransmitter Levels

#### 2.10.1. Estimation of Dopamine Level

The level of dopamine in the striatum is a marker of neuronal excitability leading to mood alterations. The dopamine levels in the brain were estimated via high-performance liquid chromatography (HPLC) using an electrochemical detector (ECD). A Waters standard system comprised of a high-pressure isocratic pump, a 20-microlitre manual sample injector valve, a C18 reversed-phase column, and an ECD was used in the study. The mobile phase is comprised of sodium citrate buffer (pH 4.5)–acetonitrile (87:13, *v*/*v*). The sodium citrate buffer contained 10 mM citric acid, 25 mM sodium hydroxide, 25 mM EDTA (ethylene diamine tetraacetic acid), and 2 mM 1-heptane sulfonic acid. The electrochemical conditions for the experiment were +0.75 V, with sensitivity ranging from 5 to 50 nA. The separation was carried out at a 0.8 mL/minute flow rate. The samples (20 microlitres) were injected manually. On the experiment day, the frozen brain samples were thawed and homogenized in a homogenizing solution containing 0.2 M perchloric acid. Then, the samples were centrifuged at 12,000× *g* for 5 min. The supernatant was filtered through 0.22 mm nylon filters before being injected into the HPLC sample injector. Data were recorded and analysed with Breeze software. The dopamine activity in rat brain homogenates is expressed as ng/mg protein [63].

#### 2.10.2. Assessment of Brain Glutamate Level

Glutamate was quantified after the derivatization with o-phthalaldehyde/β- mercaptoethanol (OPA/β-ME), filtered through 0.22 mm nylon filters before injecting into HPLC injectors. The glutamate levels were estimated by the method described by Sharma and his co-workers with slight modifications, which involved HPLC with an ECD. A Waters standard system comprised of a high-pressure isocratic pump, a 20-microlitre manual sample injector valve, a C18 reversed-phase column, and an ECD was used in the study. The mobile phase comprised 100 mM anhydrous disodium hydrogen phosphate, 25 mM EDTA, and 22% methanol (pH: 6.5). The electrochemical conditions for the experiment were +0.65 V, with sensitivity ranging from 5 to 50 nA. The separation was carried out at a 1.2 mL/minute flow rate, and the column temperature was maintained at 40 °C. Samples (20 μL) were injected manually through a rheodyne valve injector. On the day of the experiment, the frozen brain samples were thawed and homogenized in 0.2 M perchloric acid. Then, the samples were placed anteroposterior at 12,000× *g* for 15 min. The supernatant was derivatized using OPA/β-ME (o-pthalaldehyde/β-mercaptoethanol) and filtered through the 0.22 mm nylon filters before being injected into the HPLC sample injector. Data were recorded and analyzed using Breeze software version 3.2 purchases from Waters HPLC. The amino acid concentrations were calculated from the standard curve generated using a standard in 10–100 ng/mL concentration range. The glutamate activity in rat brain homogenates is expressed as ng/mg protein [57].

#### 2.10.3. Measurement of Acetylcholine (Ach) Level

ACH level was measured using a diagnostic kit (E-EL-0081/acetylcholine; Elabsciences, Wuhan, Hubei, China). The level was measured in the sample of brain homogenate by following all the instructions provided by the manufacturer. The micro ELISA plate provided in this kit has been pre-coated with ACH. During the reaction, ACH in the sample or standard competes with a fixed amount of ACH on the solid phase supporter for sites on the Biotinylated Detection Ab specific to ACH. Excess conjugate and unbound samples or standard washed from the plate, and Avidin conjugated to Horseradish Peroxidase (HRP) were added to each microplate well and incubated. Then a TMB substrate solution was added to each well. The enzyme-substrate reaction was terminated by adding a stop solution, and the colour change was measured spectrophotometrically at a wavelength of 450 nm ± 2 nm. The concentration of ACH in the samples was determined by comparing the OD of the samples to the standard curve. Tissues should be minced into small pieces and rinsed in ice-cold PBS (0.01 M, pH = 7.4) to remove excess blood thoroughly. Tissue pieces should be weighed and then homogenized in PBS (tissue weight (g): PBS (mL) volume = 1:9) with a glass homogenizer on ice. Sonication of the suspension with an ultrasonic cell disrupter was done. The homogenates were then centrifuged for 5 min at 5000× *g* to get the supernatant. Brought all reagents to room temperature (18~25 °C) before use. Followed the microplate reader manual for set-up and preheated it for 15 min before OD measurement. To prepare 750 mL of wash buffer, diluted 30 mL of concentrated wash buffer with 720 mL of deionized or distilled water. Note: if crystals have formed in the concentrate, warm it in a 40 °C water bath and mix it gently until the crystals have completely dissolved. Centrifuged the standard at 10,000× *g* for 1 min. Added 1.0 mL of reference standard and sample diluent, let it stand for 10 min and inverted it gently several times. A pipette was used to mix it thoroughly after it had dissolved. This reconstitution produced a working solution of 1000 pg/mL. Then as per the need, serial dilutions were made. The recommended dilution gradient is as follows: 1000, 500, 250, 125, 62.5, 31.25, 15.63, 0 pg/mL. Dilution method: Take 7 EP tubes, add 500 uL of Reference Standard and Sample Diluent to each tube. Pipette 500 uL of the 1000 pg/mL stock solution to the first tube and mix up to produce a 500 pg/mL working solution. According to these steps, pipette 500 uL of the solution from the former tube into the latter one. The illustration below is for reference. Note: the last tube is regarded as a blank. Do not pipette solution into it from the former tube. Calculate the required amount before the experiment (50 μL/well). The preparation should be made slightly more than calculated. Centrifuge the stock tube before use, dilute the 100× Concentrated Biotinylated Detection Ab to 1× working solution with Biotinylated Detection Ab Diluent. Calculate the required amount before the experiment (100 μL/well). The preparation should be prepared slightly more than calculated. Dilute the 100× Concentrated HRP Conjugate to 1× working solution with Concentrated HRP Conjugate Diluent. Add the Standard working solution to the first two columns: Each solution concentration is added in duplicate to one well each, side by side (50 uL for each well). Add the samples to the other wells (50 uL for each well). Immediately add 50 μL of Biotinylated Detection Ab working solution to each well. Cover the plate with the sealer provided in the kit. Incubate for 45 min at 37 °C. Note: Solutions should be added to the bottom of the micro ELISA plate well to avoid touching the inside wall and causing foaming as much as possible. Aspirate or decant the solution from each well, add 350 uL of wash buffer to each well. Soak for 1~2 min, aspirate or decant the solution from each well, and pat it dry against clean absorbent paper. Repeat this wash step 3 times. Note: a microplate washer can be used in this step and other wash steps. Add 100 μL of HRP Conjugate working solution to each well. Cover with the Plate sealer. Incubate for 30 min at 37 °C. Aspirate or decant the solution from each well, repeat the wash process five times as conducted in step 2. Add 90 μL of substrate reagent to each well. Cover with a new plate sealer. Incubate for about 15 min at 37 °C. Protect the plate from light. Note: the reaction time can be shortened or extended according to the actual colour change, but not more than 30 min. Add 50 μL of Stop Solution to each well. Note: The addition of the stop solution should be done in the same order as the substrate solution. Determine the optical density (OD value) of each well at once with a micro-plate reader set to 450 nm. Average the duplicate readings for each standard and sample. Plot a four-parameter logistic curve on log-log graph paper, with standard concentration on the x-axis and OD values on the y-axis. If the samples have diluted, the concentration calculated from the standard curve must be multiplied by the dilution factor. If the OD of the sample is under the lowest limit of the standard curve, you should re-test it with an appropriate dilution. The actual concentration is the calculated concentration multiplied by the dilution factor. The optical density of the reaction mixture was determined at 540 nm in the microtiter plate. The neurotransmitter in the supernatant was expressed as ng/mg protein [59].

#### 2.10.4. Measurement of Serotonin Level

Serotonin levels were estimated in the brain homogenate sample by HPLC using an electrochemical detector and C18 reverse-phase column. The procedure was performed according to the method described by Kumar and their co-workers. In brief, the mobile phase consisting of sodium citrate buffer (pH 4.5)–acetonitrile (87:13, *v*/*v*) was prepared. The electrochemical conditions for the experiment were +0.75 V, with sensitivity ranging from 5 to 50 nA. The separation was carried out at a 0.8 mL/min flow rate. Sample (20 μL) was injected manually after filtration through 0.22 mm nylon filters. The data were then analysed using breeze software. The standard curve generated with a concentration of 10–100 mg/mL was used to estimate the level of serotonin. The values were expressed as ng/mg protein [42].

### 2.11. Assessment of Inflammatory Cytokines

#### 2.11.1. Measurement of Tumor Necrotic Factor-Alpha (TNF-α) Level

The level of TNF-α were quantified by using rat TNF-α immunoassay kit (E-EL-R0019/TNF-α; ELabSciences, Wuhan, Hubei, China). The activity level of TNF-α was quantified in brain homogenate [59] and blood plasma [8] according to the instructions provided by the manufacturer. It is a solid-phase sandwich enzyme-linked immunosorbent assay (ELISA), which uses a microtitre plate reader read at 450 nm. Concentrations of TNF-α were calculated from the plotted standard curve. This assay employs the quantitative sandwich enzyme immunoassay technique. An affinity polyclonal antibody specific for mouse TNF-α has been pre-coated onto a microplate. Standards, controls and samples are pipette into wells and the immobilized antibody binds any rat TNF-α present. After washing away any unbound substances, an enzyme-linked polyclonal antibody specific for rat TNF-α is added to the wells. Following a wash to remove any unbound antibody-enzyme reagent, a substrate solution is added to the wells. The enzyme reaction yields a blue product that turns yellow when the stop solution is added. The intensity of the colour measured is in proportion to the amount of rat TNF-α bound in the initial step. The sample values are then read off the standard curve. The reagent and standard dilutions were prepared as suggested by the manufacturer’s instructions. An amount of 50 μL of assay diluents were added to each well of the pre-coated microtitre plate. Then, 50 μL of standard or sample was added per well and mixed gently. The plate was covered with an adhesive strip and incubated for 2 h at room temperature. Each well was then aspirated and washed with wash buffer, repeating the process four times for a total of five washes. The plate was then inverted and blotted against a clean paper towel. Next, 100 μL of rat Aβ-conjugate was added to each well and covered with a new adhesive strip. The plate was again incubated for 2 h at room temperature. The aspiration/washes were repeated as in step 5. One hundred microlitres of substrate solution was then added to each well and incubated for 30 min at room temperature, protected from light. Finally, 100 μL of stop solution was added to each well, and the absorbance of each well was read in an ELISA reader set to 450 nm. The average for the triplicate readings was calculated for each standard and sample, and the blank values were subtracted. A standard curve was constructed by plotting the mean absorbance for each standard on the y-axis against the concentration on the *x*-axis, and the best fit curve was drawn through the points on the graph by regression analysis. The diluted samples were multiplied by the dilution factor to get the exact concentration of the unknown samples. The activity of TNF-α in rat brain homogenate was expressed as pg/mg protein.

#### 2.11.2. Measurement of Interleukin-1 Beta (IL-1β) Level

The level of IL-1β was quantified by using a rat IL-1β immunoassay kit (E-EL-R0012/IL-1β; ELabSciences, Wuhan, Hubei, China). The level of IL-1β has also been measured in rat’s brain homogenate [61] and blood plasma [72] according to the instructions provided by the manufacturer. It is a solid-phase sandwich enzyme-linked immunosorbent assay (ELISA), which uses a microtitre plate reader read at 450 nm. Concentrations of IL-1β were calculated from the plotted standard curve. This assay employs the quantitative sandwich enzyme immunoassay technique. Antibody specific for Interleukin 1 beta (IL-1β) has been pre-coated onto a microplate. Standards and samples were pipetted into the wells, and any Interleukin 1 beta (IL-1β) present is bound by the immobilized antibody. After removing any unbound substances, a biotin-conjugated antibody specific to the wells was added to Interleukin 1 beta (IL-1β). After washing, avidin conjugated was added to the wells. Following a wash to remove any unbound avidin-enzyme reagent, a substrate solution was added to the wells and colour developed in proportion to the amount of Interleukin 1 beta (IL-1β) bound in the initial step. The colour development was stopped, and the intensity of the colour was measured. The reagent and standard dilutions were prepared as suggested by the manufacturer’s instructions. An amount of 100 μL of assay diluents was added to each well of the pre-coated microtitre plate. Then, 100 μL of standard or sample was added per well and mixed gently. The plate was covered with an adhesive strip and incubated for 2.5 h at room temperature. Each well was then aspirated and washed with wash buffer, repeating the process four times for a total of five washes. The plate was then inverted and blotted against a clean paper towel. Next, 100 μL of rat biotinylated antibody was added to each well and covered with a new adhesive strip. The plate was again incubated for 1 h at room temperature with gentle shaking. The aspiration/washes were repeated as in step 5. One hundred microlitres of streptavidin solution was added to each well and incubated for 45 min at room temperature, protected from light. The solution was discarded. The wash was repeated as in step 5. Then, 100 μL of TMB one-step substrate reagent was added to each well and incubated for 30 min at room temperature. Last, 50 μL of stop solution was finally added to each well, and the absorbance of each well was read in an ELISA reader set to 450 nm. The average for the triplicate readings was calculated for each standard and sample, and the blank values were subtracted. A standard curve was constructed by plotting the mean absorbance for each standard on the *y*-axis against the concentration on the *x*-axis, and the best fit curve was drawn through the points on the graph by regression analysis. The samples which were diluted were multiplied by the dilution factor to get the exact concentration of the unknown samples. The measured levels are expressed as pg/mg protein in brain homogenate and pg/mL in blood plasma.

### 2.12. Assessment of Oxidative Stress Markers

#### 2.12.1. Estimation of Acetylcholinesterase (AChE) Level

The assay mixture contained 0.05 mL of supernatant of brain homogenate, 3 mL of 0.01 M sodium phosphate buffer (pH 8), 0.10 mL of acetylthiocholine iodide, and 0.10 mL DTNB (Ellman reagent). The change in absorbance was measured using a UV spectrophotometer at a wavelength of 412 nm. The values were expressed as µM/mg protein [73].

#### 2.12.2. Measurement of Superoxide Dismutase (SOD) Enzymatic Activity

SOD activity was measured according to the method performed by [74] spectrophotometrically. The mixture of 3 samples was prepared, including 0.02 mL epinephrine, 0.2 mL supernatant of the brain homogenate, and 0.8 mL of 50 mM glycine buffers, pH 10, and the reaction was started. After 5 min, the absorbance was measured spectrophotometrically using a UV spectrophotometer at 480 nm. The activity of SOD was expressed as nM/mg protein [74].

#### 2.12.3. Estimation of Lactate Dehydrogenase (LDH) Level

Lactate dehydrogenase levels in rat brain homogenate were determined using a diagnostic kit from Coral Diagnostics, Goa, India, and are reported as unit/mg protein [73].

#### 2.12.4. Estimation of Malondialdehyde (MDA) Level

The MDA level was measured by reacting it with thiobarbituric acid; the MDA amount was measured at 532 nm using a spectrophotometer. The concentration of MDA was expressed as nM/mg protein [60].

#### 2.12.5. Estimation of Reduced Glutathione Level

To measure glutathione level, the precipitation was carried out using 1 mL of 4% sulfosalicylic acid with 1 mL supernatant of brain homogenate and cold digested at 4 °C for one hour. The brain homogenate sample was centrifuged at 1200× *g* for 15 min. After that, 2.7 mL of phosphate buffer (0.1 M, pH 8) and 0.2 mL of 5,5′-dithiobis-(2-nitrobenzoic acid) (DTNB) were added to the 1 mL of supernatant. The yellow colour was developed, and immediately absorbance was taken at 412 nm using a spectrophotometer. The values were expressed as µM/mg protein [75].

#### 2.12.6. Assessment of Nitrite Level

A colorimetric test employing Greiss reagent (0.1% N-(1-naphthyl) ethylenediamine dihydrochloride, 1% sulfanilamide, and 2.5% phosphoric acid) determines the buildup of nitrite in the supernatant of brain homogenate, which is an indication of the generation of nitric oxide (NO). Equal quantities of supernatant and Greiss reagent are combined, the mixture is incubated in the dark for 10 min at room temperature, and the absorbance is measured spectrophotometrically at 540 nm. A sodium nitrite standard curve calculates the nitrite concentration in the supernatant, represented as µM/mg protein [76].

### 2.13. Assessment of Gross Pathology and Morphology

Animals were sacrificed on day 45 of the protocol schedule by decapitation; brains were removed for gross pathological analysis. After observing the whole rat brain, 2-mm thick coronal sections (coronally from the anterior pole to the posterior cerebral cortex poles) of the fresh brain were taken [54]. These coronal sections were then mounted on a glass slide to visualize all the brain areas, a digital camera (Fujix digital camera, Fujifilm, Yokyo, Japan) was utilized. The digital images obtained were analysed and converted into TIFF files for further analysis. The damaged and demyelination region (mm) in each brain segment was observed through MOTICAM-BA310 image plus 2.0 analysis software. The demyelination scale (mm) volume was calculated for each coronal brain segment by converting the demyelination region (mm). The dark greyish area in the brain sections of the basal ganglia region depicts the demyelination region, and the demyelination size (mm^3^) in each brain section was determined on the 45th day. The magnitude of the damage was determined by calculating the demyelination area (l × b × h) in each coronal 2-mm-thick brain slice [63].

### 2.14. Assessment of Histopathological Changes

The same group of animals was maintained for histological studies. The brains were removed from the skull; the cerebral cortex region was isolated from the whole brain, cleaned, and sliced into 0.5 cubic cm. After further fixation by immersion in 4% paraformaldehyde in PBS pH 7.4 overnight at RT (8–12 h), washing in PBS and immersion in 70% ethanol was done. The tissue was maintained at 37 y immersion in 4% paraformaldehyde in PBS pH 7.4 overnight at RT (8–12 h), washed in PBS, and immersed in 70% ethanol. The tissue was maintained at 37 °C until the embedding in paraffin was made. The paraffin blocks were cut into 10 µm thickness using a rotary microtome. The sections were stained with haematoxylin and eosin, and the morphology was analysed by fluorescence microscope (typeM) (100× magnification). The density of normal neuronal population (pyramidal cells, stellate cells, oligodendrocytes, astrocytes, and microglial cells) of all cerebral cortex regions was counted at a defined coronal level in a blinded fashion with reticule incorporated eyepiece at a magnification of ×100 using a fluorescence microscope. Using anatomical landmarks, a total of eight sections of similar levels of amygdale within and between control and experimental groups were analysed with the optical microscope.

### 2.15. Statistical Analysis

Data were analysed using two-way ANOVA followed by a post hoc test Bonferroni and one-way ANOVA repeated measures followed by a post hoc Tukey’s multi comparison test. *p* < 0.001 was considered statistically significant. Data were found to be normalized, and the sample size was calculated by checking the normality distribution by the Kolmogorov Smirnov test. All statistical results were performed using GraphPad Prism version 5.03 for Windows (GraphPad Software, San Diego, CA, USA). Statistical results were expressed as the mean ± standard error of the mean (SEM).

## 3. Results

### 3.1. Neuroprotective Potential of Guggulsterone in the Restoration of Weight Variations in Propionic Acid-Induced Experimental Model of Autism in Adult Rats

#### 3.1.1. Improvement in Body Weight with Long-Term Guggulsterone Treatment

**Figure 2:**
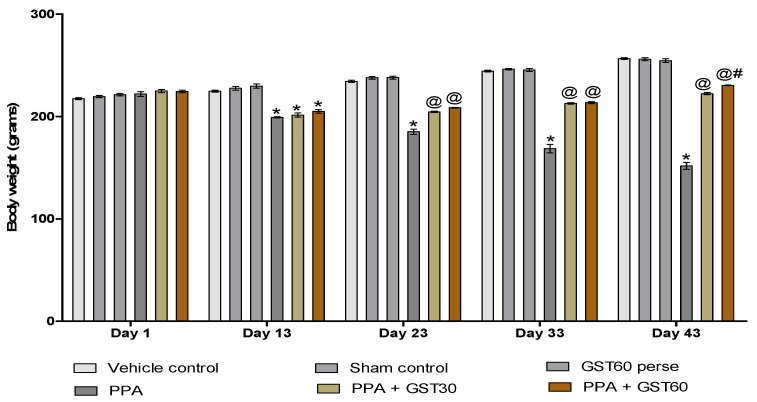
Neuroprotective potential of guggulsterone on bodyweight in propionic acid-induced experimental model of autism in adult rats. Statistical analysis followed by two-way ANOVA (post hoc Bonferroni test). Values are expressed as mean ± SEM (*n* = 6 rats per group).* *p* < 0.001 v/s normal control, vehicle control and GST60 *perse*; @ *p* < 0.001 v/s PPA; @# *p* < 0.001 v/s PPA + GST30.

Alterations in body weight were assessed between all six groups on the experimental protocol schedule’s 1st, 13th, 23rd, 33rd, and 43rd days. None of the experimental groups showed any significant changes in body weight on the 1st day. Regular ICV-injection of PPA was given for the first 11 days, which led to a decrease in the bodyweight of the treated groups. On the 13th day, PPA-treated groups significantly decreased body weight compared to vehicle, sham, and GST60 perse treated groups (*p* < 0.001). After chronic oral administration of GST30 mg/kg and GST60, mg/kg body weight was substantially restored on days 23, 33, and 43 as compared to the PPA-induced experimental model of autism in adult rats [two way ANOVA: F(20,120) = 186.5, *p* < 0.001]. On the 43rd day, GST60 mg/kg treated rat groups revealed a significant dose-dependent bodyweight improvement (Figure 2).

#### 3.1.2. Improvement in the Relative Brain-Body Weight Ratio with Long Term Guggulsterone Treatment

**Figure 3:**
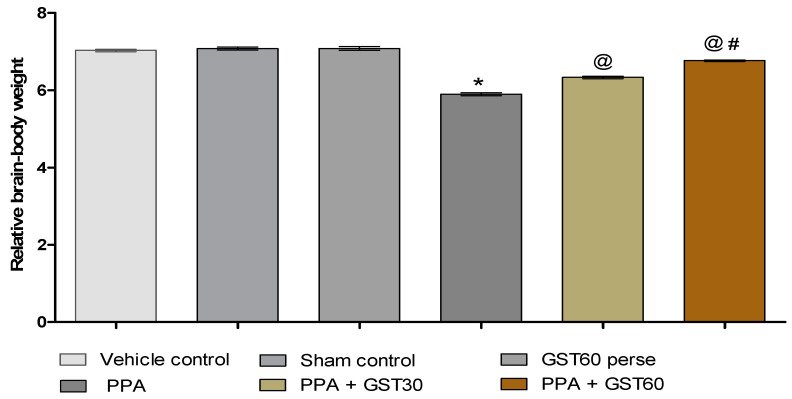
Neuroprotective potential of guggulsterone on relative brain-body weight ratio in propionic acid-induced experimental model of autism in adult rats. Statistical analysis followed by one-way ANOVA (post hoc Tukey’s test). Values are expressed as mean ± SEM (*n* = 6 rats per group). * *p* < 0.001 v/s normal control, vehicle control and GST60 *perse*; @ *p* < 0.001 v/s PPA; @# *p* < 0.001 v/s PPA + GST30.

The relative brain-body weight ratio was assessed at the end of the experimental protocol schedule. Compared to the vehicle, sham, and GST60 perse groups, PPA-treated autistic rats significantly decreased brain-body weight ratio (*p* < 0.001). Interestingly, GST30 and GST60 mg/kg groups showed a substantial dose-dependent restoration in the relative-brain body weight ratio when compared to the PPA-induced experimental model of the autism group [one-way ANOVA: F(5,25) = 0.725, *p* < 0.001] (Figure 3).

### 3.2. Neuroprotective Potential of Guggulsterone in the Prevention of Neurobehavioral Impairments in Propionic Acid-Induced Experimental Model of Autism in Adult Rats

#### 3.2.1. Improved Locomotor Activity with Long Term Guggulsterone Treatment

**Figure 4:**
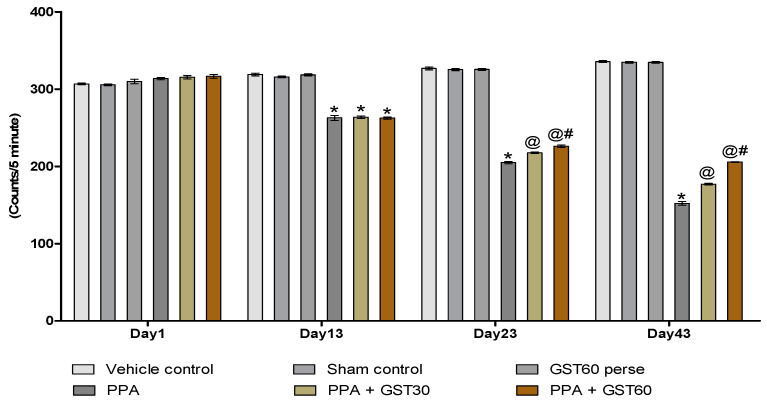
Neuroprotective potential of guggulsterone on locomotor activity in propionic acid-induced experimental model of autism in adult rats. Statistical analysis followed by two-way ANOVA (post hoc Bonferroni test). Values are expressed as mean ± SEM (*n* = 6 rats per group). * *p* < 0.001 v/s normal control, vehicle control and GST60 *perse*; @ *p* < 0.001 v/s PPA; @# *p* < 0.001 v/s PPA + GST30.

Actophotometer was used to observe the locomotor activity on the experimental protocol schedule’s 1st, 13th, 23rd, and 43rd days. On day 1st, there was no remarkable difference in locomotion between all the experimental groups. On the 13th day, the PPA-treated experimental group showed a significant change in locomotion compared to the vehicle, sham, and GST60 perse treated rats (*p* < 0.001). On 23rdday, GST30 mg/kg and GST60 mg/kg experimental groups exhibit improvement in locomotor activity when compared to PPA-treated autistic rats [two way ANOVA: F(15,90) = 545.9, *p* < 0.001]. On the 23rd and 43rd days, there was a significant difference in locomotor activity between the GST30 mg/kg and GST60 mg/kg groups, indicating a dose-dependent association between motor activity and GST (Figure 4).

#### 3.2.2. Improvement in Cognitive and Memory with Long Term Guggulsterone Treatment

**Figure 5:**
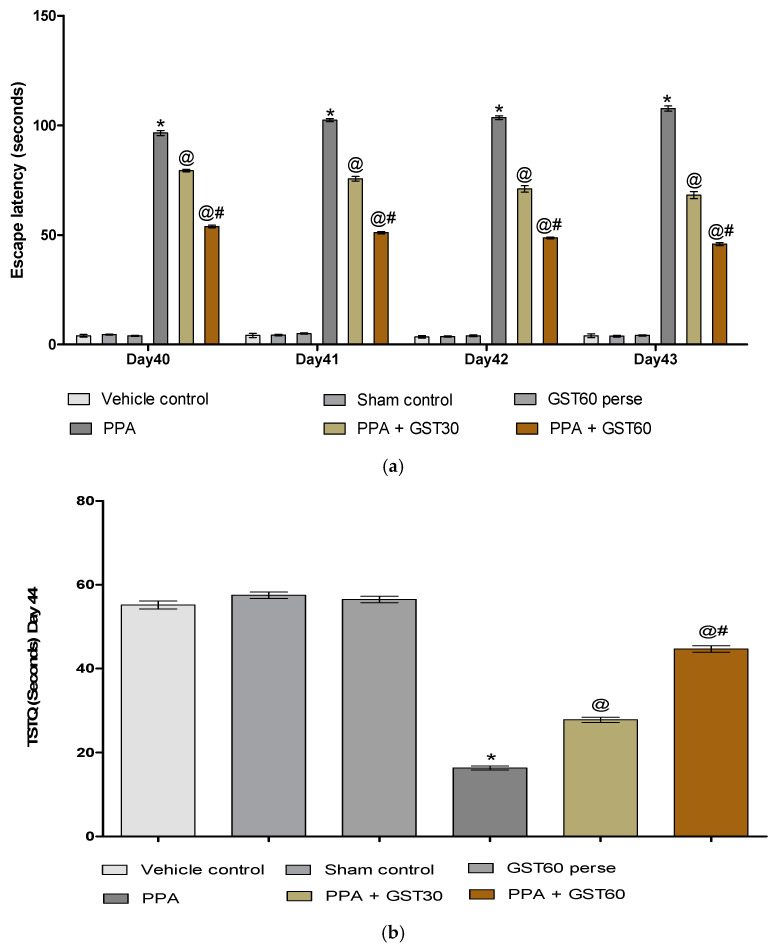
(**a**). Neuroprotective potential of guggulsterone on escape latency time in propionic acid-induced experimental model of autism in adult rats. Statistical analysis followed by two-way ANOVA (post hoc Bonferroni test). Values are expressed as mean ± SEM (*n* = 6 rats per group). * *p* < 0.001 v/s normal control, vehicle control and GST60 *perse*; @ *p* < 0.001 v/s PPA; @# *p* < 0.001 v/s PPA + GST30. (**b**). Neuroprotective potential of guggulsterone on TSTQ in propionic acid-induced experimental model of autism in adult rats. Statistical analysis followed by one-way ANOVA (post hoc Tukey’s test). Values are expressed as mean ± SEM (*n* = 6 rats per group). * *p* < 0.001 v/s normal control, vehicle control and GST60 *perse*; @ *p* < 0.001 v/s PPA; @# *p* < 0.001 v/s PPA + GST30.

On the 40th, 41th, 42nd, and 43rd days, escape latency time (ELT) was evaluated using the Morris water maze. TSTQ was monitored according to the experimental protocol schedule on day 44th. PPA-induced autistic rats show a substantial rise in ELT compared to the vehicle, sham, and GST60 perse groups. Chronic administration of GST30 mg/kg and GST60 mg/kg eventually reduced ELT when compared to PPA-induced autistic group [two-way ANOVA: F(15,90) = 32.43, *p* < 0.001]. GST60 mg/kg treated rats showed a dose-dependent improvement in long-term memory and cognition compared to GST30 mg/kg treated rats (Figure 5a).

The PPA-induced experimental model of autism in adult rats showed a significant reduction in TSTQ compared to the vehicle, sham, and GST60 perse groups. However, as compared to autistic treated rats, oral administration of GST30 mg/kg and GST60 mg/kg resulted in a significant increase in TSTQ [one-way ANOVA: F(5,25) = 2.766, *p* < 0.001]. As the protocol schedule was completed, it was found that the GST60 mg/kg treated group was more successful and showed a dose-dependent restoration in consolidating memory when compared to the GST30 mg/kg treated group (Figure 5b).

#### 3.2.3. Recovery of Muscle Coordination with Long Term Guggulsterone Treatment

**Figure 6:**
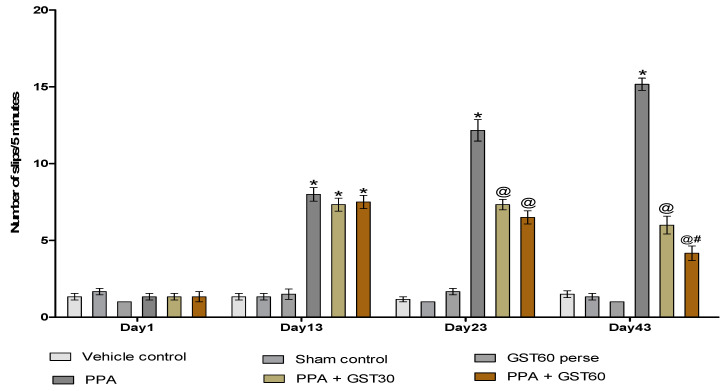
Neuroprotective potential of guggulsterone on number of slips in propionic acid-induced experimental model of autism in adult rats. Statistical analysis followed by two-way ANOVA (post hoc Bonferroni test). Values are expressed as mean ± SEM (*n* = 6 rats per group). * *p* < 0.001 v/s normal control, vehicle control and GST60 *perse*; @ *p* < 0.001 v/s PPA; @# *p* < 0.001 v/s PPA + GST30.

Neuromuscular coordination was observed by performing a BCT on the experimental protocol schedule’s 1st, 13th, 23rd, and 43rd days. On the 1st day, there was no significant difference between all the treated groups. On the 13th day of the experimental protocol schedule, PPA-treated autistic rats showed a remarkable increase in the number of slips compared to vehicle, sham, and GST60 perse groups (*p* < 0.001). On the 23rd and 43rd days of the experimental protocol schedule, there was a decrease in the number of slips after the chronic administration of GST30 mg/kg and GST60 mg/kg treated rats [two-way ANOVA: F(15,90) = 64.54, *p* < 0.001]. It was also shown that on the 43rd day, the GST60 mg/kg group had a significant decrease in the number of slips compared to the GST30 mg/kg group, indicating that GST has a dose-dependent effect in the recovery of neuromuscular coordination (Figure 6).

#### 3.2.4. Improvement in Depression-like Behaviour with Long Term Guggulsterone Treatment

**Figure 7:**
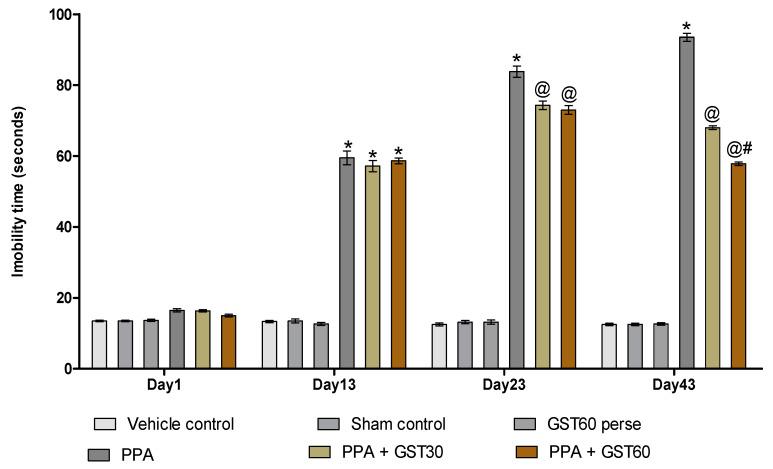
Neuroprotective potential of guggulsterone on immobility time in propionic acid-induced experimental model of autism in adult rats. Statistical analysis followed by two-way ANOVA (post hoc Bonferroni test). Values are expressed as mean ± SEM (*n* = 6 rats per group). * *p* < 0.001 v/s normal control, vehicle control and GST60 *perse*; @ *p* < 0.001 v/s PPA; @# *p* < 0.001 v/s PPA + GST30.

The immobility time was measured using a forced swim test on the 1st, 13th, 23rd, and 43rd day of the experimental protocol schedule. There was no remarkable variation in immobility time on the first day among any experimental groups. On the 13th day of the experimental protocol schedule, PPA-treated rats showed a substantial increase in immobility time compared to the vehicle, sham, and GST60 perse groups (*p* < 0.001). There was a significant reduction in the immobility time after the chronic administration of GST30 mg/kg and GST60 mg/kg on 23rd and 43rddays when compared to the PPA-induced experimental model of autism in adult rats [two-way ANOVA: F(15,90) = 472.7, *p* < 0.001]. On the 43rd day of the experimental procedure schedule, the GST60 mg/kg group demonstrated a significant decrease in immobility time compared to the GST30 mg/kg group. This finding suggests a dose-dependent relationship between GST and immobility time (Figure 7).

### 3.3. Neuroprotective Potential of Guggulsterone on Neurochemical Alterations in Propionic Acid-Induced Experimental Model of Autism in Adult Rats

#### 3.3.1. Decreased STAT3 Level with Long-Term Guggulsterone Treatment

The level of STAT3 protein in brain homogenate and cerebrospinal fluid (CSF) was evaluated at the end of the experimental protocol schedule. In brain homogenate and CSF, the level of STAT3 was significantly higher in PPA-treated rats than in the vehicle, sham, and GST60 perse groups (*p* < 0.001). Chronic treatment with GST30 mg/kg and GST60 mg/kg showed a decrease in the level of STAT3 protein in brain homogenate [one-way ANOVA: F(5,25) = 6.468, *p* < 0.001] and CSF [one-way ANOVA: F(5,25) = 2.113, *p* < 0.001] when compared to PPA-induced experimental group. GST60 mg/kg treated group significantly showed greater effectiveness in reducing STAT3 when compared GST30 mg/kg treated group (Table 1).

#### 3.3.2. Increased PPAR-Gamma Level with Long Term Guggulsterone Treatment

At the end of the experimental protocol, the level of PPAR-gamma protein in brain homogenate and CSF was measured. Compared to the vehicle, sham, and GST60 perse groups, PPA-treated autistic rats significantly decreased PPAR-gamma levels in brain homogenate and CSF (*p* < 0.001). Long-term treatment with GST30 mg/kg and GST60 mg/kg resulted in a significant rise in the level of PPAR gamma protein in brain homogenate [one-way ANOVA: F(5,25) = 1.616, *p* < 0.001] and CSF [one-way ANOVA: F(5,25) = 0.266, *p* < 0.001] as compared to the PPA-induced autism group. GST60 mg/kg was more effective in increasing PPAR gamma levels than GST30 mg/kg treated rat groups among different treatment doses (Table 1).

#### 3.3.3. Increased Myelin Basic Protein Level with Long Term Guggulsterone Treatment

To study the effect of PPA and GST on rat’s brain, myelin basic protein level was evaluated at the end of the experimental protocol schedule in brain homogenate. The level of myelin basic protein in PPA-treated rats was considerably lower than in the sham, vehicle, and GST60 perse groups (*p* < 0.001). Myelin’s basic protein levels in brain homogenate were considerably higher after prolonged treatment with GST30 mg/kg and GST60 mg/kg than PPA-induced rats [one-way ANOVA: F(5,25) = 0.576, *p* < 0.001]. GST60 mg/kg was more efficient in restoring myelin basic protein levels than GST30 mg/kg (Table 1).

#### 3.3.4. Decreased Level of Caspase-3, Bax, and Increased Level of Bcl-2 with Long Term Guggulsterone Treatment

At the end of the experimental protocol, various apoptotic biomarkers such as Bax, Caspase-3, and Bcl-2 were evaluated in brain homogenate and blood plasma. Bax and Caspase-3 protein levels increased following PPA administration compared to the vehicle, sham, and GST60 perse groups; however, Bcl-2 protein levels were reduced in both brain homogenate and blood plasma (*p* < 0.001). Long-term treatment with GST30 mg/kg and GST60 mg/kg remarkably reduce apoptosis by decreasing the level of Caspase-3 [one-way ANOVA: F(5,25) = 2.256, *p* < 0.001] and Bax [one-way ANOVA: F(5,25) = 1.809, *p* < 0.001]. The level of Bcl-2 [one-way ANOVA: F(5,25) = 4.729, *p* < 0.001] was found to have increased after GST treatment. GST60 mg/kg was shown to be more effective than GST30 mg/kg in modifying the level of apoptotic markers in brain homogenate among different treatment doses (Table 2).

In blood plasma, GST treatment at different doses (30 and 60 mg/kg) was also found to significantly modulate the level of apoptotic markers. In comparison with GST30 mg/kg, GST60 mg/kg had more considerable effect in decreasing the level of Caspase-3 [one-way ANOVA: F(5,25) = 1.133, *p* < 0.001] and Bax [one-way ANOVA: F(5,25) = 1.653, *p* < 0.001] and restore the level of Bcl-2 [one-way ANOVA: F(5,25) = 1.098, *p* < 0.001] (Table 2).

#### 3.3.5. Restoration of Neurotransmitter Levels after Long Term Treatment with Guggulsterone

To investigate guggulsterone’s neuroprotective potential, neurotransmitters such as serotonin, glutamate, dopamine, and acetylcholine were evaluated in rat brain homogenate at the end of the experimental protocol schedule. PPA-induced experimental rats had substantially higher levels of glutamate and lower levels of serotonin, dopamine, and acetylcholine compared to the sham group, vehicle group, and GST60 perse group (*p* < 0.001).

After chronic treatment with GST30 mg/kg and GST60 mg/kg, it was found that level of glutamate [one-way ANOVA: F(5,25) = 2.045, *p* < 0.001] was considerably decreased, whereas levels of serotonin [one-way ANOVA: F(5,25) = 2.203, *p* < 0.001], dopamine [one-way ANOVA: F(5,25) = 3.731, *p* < 0.001] and acetylcholine [one-way ANOVA: F(5,25) = 5.135, *p* < 0.001] was significantly increased in the brain homogenate. GST60 mg/kg was also shown to be more effective than GST30 mg/kg in restoring neurotransmitter levels, showing a dose-dependent relationship between GST and neurotransmitters (Table 3).

#### 3.3.6. Reduction in Neuroinflammatory Cytokines after Long-Term Treatment with Guggulsterone

Inflammatory cytokines (TNF-α and IL-1β) were measured in brain homogenate and blood plasma at the end of the experiment. TNF-α and IL-1β levels in brain homogenate and blood plasma were considerably higher in PPA-induced experimental rats than in the sham, vehicle, and GST60 perse groups (*p* < 0.001). When compared to PPA-treated rats, long-term treatment with GST30 mg/kg and GST60 mg/kg remarkably reduced TNF-α level in brain homogenate [one-way ANOVA: F(5,25) = 0.7765, *p* < 0.001] and blood plasma [one-way ANOVA: F(5,25) = 1.23, *p* < 0.001] (Table 4).

The level of IL-1β in brain homogenate [one-way ANOVA: F(5,25) = 0.5758, *p* < 0.001] and blood plasma [one-way ANOVA: F(5,25) = 0.5813, *p* < 0.001] was also considerably reduced when GST30 mg/kg and GST60 mg/kg treatment groups were compared to PPA-treated autistic rats. Compared to GST30 mg/kg, GST60 mg/kg demonstrated a dose-dependent impact, as it was more effective in restoring elevated levels of inflammatory cytokines (Table 4).

#### 3.3.7. Restoration of the Anti-Oxidant Level after Long-Term Treatment with Guggulsterone

Oxidative stress indicators such as acetylcholinesterase (AchE), lactate dehydrogenase (LDH), superoxide dismutase (SOD), glutathione (GSH), nitrite, and malondialdehyde (MDA) were measured in brain homogenate at the end of the experimental protocol schedule. AchE, LDH, nitrite, and MDA levels in PPA-treated experimental rats were significantly higher than in the sham, vehicle, and GST60 perse groups. In contrast, the levels of SOD and GSH were considerably reduced (Table 5).

Following chronic treatment with GST30 mg/kg and GST60 mg/kg, [one-way ANOVA: F(5,25) = 5.324, *p* < 0.001], LDH [one-way ANOVA: F(5,25) = 2.731, *p* < 0.001], nitrite [one-way ANOVA: F(5,25) = 2.633, *p* < 0.001] and MDA [one-way ANOVA: F(5,25) = 1.819, *p* < 0.001] levels were significantly reduced in a dose-dependent manner (Table 5).

However, in GST30 mg/kg and GST60 mg/kg treated groups the levels of SOD [one-way ANOVA: F(5,25) = 0.1931, *p* < 0.001] and GSH [one-way ANOVA: F(5,25) = 0.3703, *p* < 0.001] were considerably high compared to PPA-treated rats. GST60 mg/kg was observed to be more efficacious in restoring anti-oxidant level compared to GST30 mg/kg (Table 5).

### 3.4. Neuroprotective Potential of Guggulsterone in the Restoration of Gross Pathological Abnormalities in Propionic Acid-Induced Experimental Model of Autism in Adult Rats

#### 3.4.1. Improvement in Whole-Brain Alterations after Long Term Treatment with Guggulsterone

**Figure 8:**
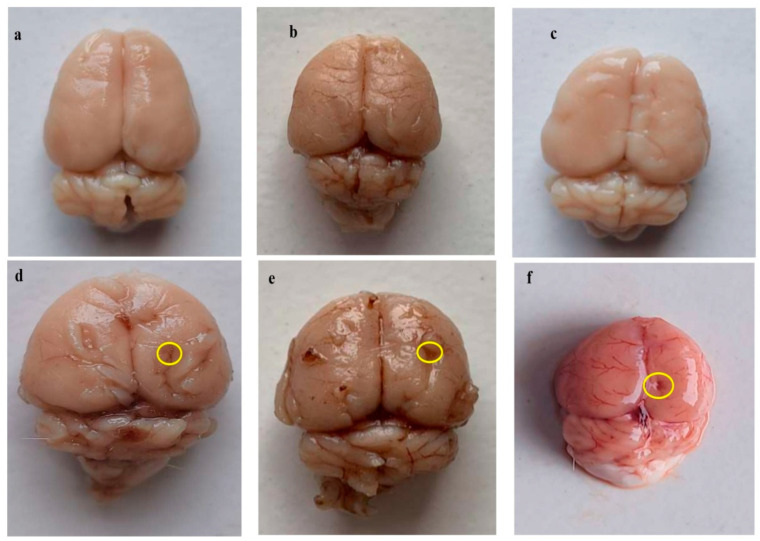
Neuroprotective potential of GST in the restoration of gross pathological alterations (Whole brain) in propionic acid-induced experimental model of autism in adult rats. (**a**) Vehicle control (**b**) Sham control (**c**) GST60 perse (**d**) PPA (**e**) PPA + GST30 (**f**) PPA+ GST60. (Scale bar = 5 mm). Note: Yellow circles are showing injury, *n* = 6 rats in each group.

After sacrificing all the animals at the end of the experimental protocol schedule, whole fresh brains were evaluated for morphological alterations. In the vehicle group, sham group, and GST60 perse group, all the brain’s normal physiological features were well preserved; however, brains isolated from PPA-treated experimental rats presented reduced total brain mass, contracted orbitofrontal cortex, degenerated meninges, and also showed a clotted outer layer. After chronic administration of GST30 mg/kg and GST60 mg/kg, substantial restoration of altered parameters and brains from both groups showed optimized size compared to the PPA-treated autistic rats group. It was also observed that GST60 mg/kg treated group showed better restoration of modified morphological features when compared to the GST30 mg/kg treated group (Figure 8).

#### 3.4.2. Reduced Pathological Abnormalities in Brain Sections after Long Term Administration of Guggulsterone

**Figure 9:**
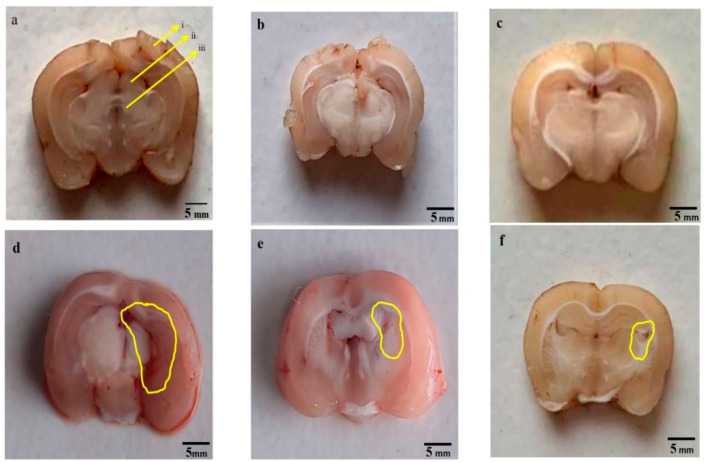
Neuroprotective potential of GST in the restoration of gross pathological alterations (brain section) in propionic acid-induced experimental model of autism in adult rats. Vehicle control: (**a**) (i) Cerebral cortex (ii) Hippocampus (iii) Basal ganglia (**b**) Sham control (**c**) GST60 *perse* (**d**) PPA (**e**) PPA + GST30 (**f**) PPA + GST60. (Scale bar = 5 mm). Note: Yellow circles are showing injury, *n* = 6 rats in each group.

All rats were sacrificed at the end of the experimental protocol, and fresh brains were separated and sectioned. Sections from PPA-treated experimental rats showed distorted basal ganglia, insula, defragmented hippocampal region; along with a reduction in cortical tissues, degenerated white matter, and myelin degeneration when compared with brain sections from the sham group, vehicle group, and GST60 perse group, which showed intact and well-defined white matter region, hippocampus and basal ganglia tissues. Chronic treatment with GST30 mg/kg and GST60 mg/kg remarkably reversed the abnormal alteration in brain sections, recomposed the reduced cortical area, restored myelin and white matter to a certain extent compared to PPA-induced experimental rats. GST 60 mg/kg compared to GST30 mg/kg was more efficacious restoring abnormal brain features (Figure 9).

#### 3.4.3. Reduced Demyelination Volume after Long Term Administration with Guggulsterone

**Figure 10:**
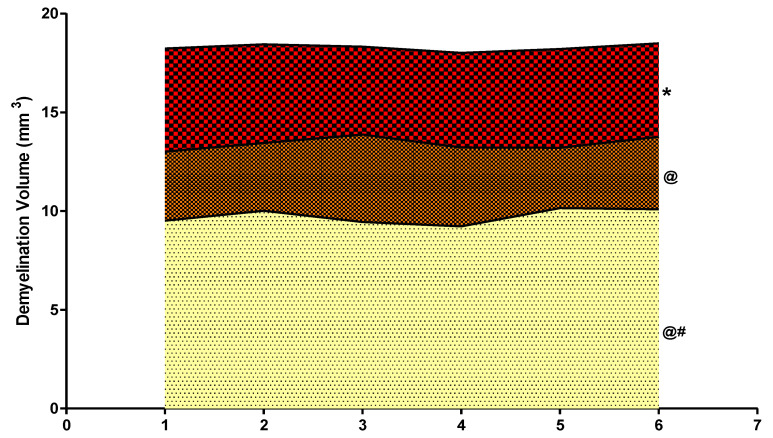
Effect of guggulsterone on demyelination volume in propionic acid-induced experimental model of autism in adult rats. Statistical analysis followed by one-way ANOVA (post hoc Tukey’s test). Values are expressed as mean ± SEM (*n* = 6 rats per group). * *p* < 0.001 v/s normal control, vehicle control and GST60 *perse*; @ *p* < 0.001 v/s PPA; @# *p* < 0.001 v/s PPA + GST30.

PPA-treated experimental rats demonstrated a significant increase in the demyelination volume compared to the vehicle, sham, and GST60 perse group, which were revealed to have zero demyelination volume. In comparison to PPA-treated autistic rats, treatment with GST at dosages of 30 mg/kg and 60 mg/kg significantly decreased demyelination volume [One-way ANOVA: F(5,25) = 1.953, *p* < 0.001]. As a result, GST60 mg/kg treated rats showed a significant, dose-dependent reduction in demyelination volume compared to GST30 mg/kg treated rats (Figure 10).

### 3.5. Neuroprotective Effect of Guggulsterone in Propionic Acid-Induced Histopathological Changes

**Figure 11:**
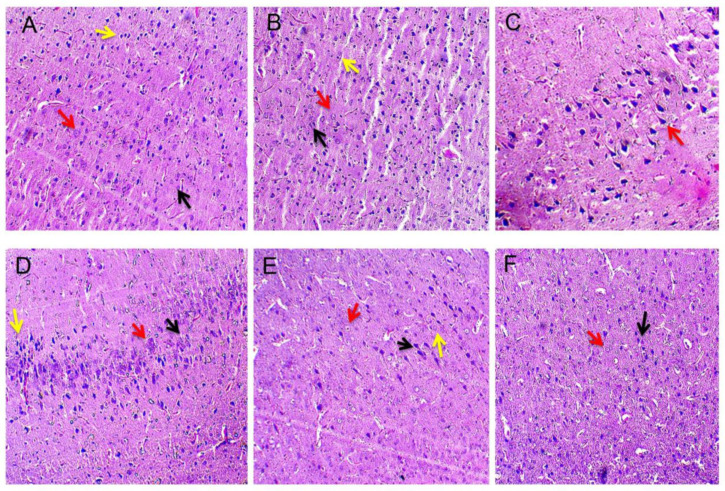
Neuroprotective effect of guggulsterone in propionic acid-induced experimental model of autism in adult rats **‘Section A’** represents the vehicle control group. The red arrow points towards the stellate cells mainly present in the cerebral cortex’s external and internal granular layer. The yellow arrow represents normal astrocytes. Prominent oligodendrocytes are seen indicated by a black arrow. **‘Section B’** represents the sham control group. The red arrow indicates healthy para-neural oligodendrocytes (often referred to as satellite cells). The yellow arrow points to the normal-appearing astrocytes. The black arrow represents rod-shaped microglial cells. **‘Section C’** depicts GST *perse* treated group. A healthy, structured, and well-defined pyramidal cell layer (red arrow) is observed with intact cytoplasm. In **‘Section D’**, degeneration of neuronal cells is visible in the propionic acid-treated brain. Structural integrity of stellate cells (red arrow) and oligodendrocytes (black arrow) is lost. In addition, the distortion of the arrangement of neuronal cells is noted. The yellow arrow represents the presence of microglial cells. Loss of pyramidal cell layer is depicted in section D. **‘Section E’** represents propionic acid, and GST (30 mg/kg) treated group in which slight regeneration of pyramidal neurons are observed indicated by the black arrow. The red arrow points to the repaired oligodendrocytes. The yellow arrow designates the presence of restored stellate cells. **‘Section F’** represents propionic acid and GST (60 mg/kg) treated group in which the group observed moderate restoration of neuronal cells. The black arrow indicates the restored structure of astrocytes. Red arrows depict the regenerated oligodendrocytes upon administration of GST. *Scale: All figures. The 11 panels are of H&E stained sections. Final magnifications (A to F) = 100×*.

Cerebral cortex neurons in the vehicle control, sham control, and GST perse treated groups displayed normal ultrastructure. These clusters contained rod-shaped microglial cells as well as healthy stellate cells, oligodendrocytes, and astrocytes. Propionic acid-treated brains exhibited neuronal cell disruption, as well as alterations in shape, size, and structure. Cytoplasmic shrinkage has also been observed. The administration of propionic acid in addition to GST (30 mg/kg) resulted in a progressive repair of the pyramidal cell layer. There was also an improvement in the structure of oligodendrocytes. GST (60 mg/kg) restored the shape of oligodendrocytes and astrocytes while keeping the cytoplasm completely intact (Figure 11).

## 4. Discussion

Autism is a neurodevelopmental condition characterized by dysfunction in three aspects: social interaction, aggressiveness, and repetitive behaviour [77]. The current work sought to investigate the protective effect of STAT3 and PPAR gamma modulator GST on restoring behavioural and neurochemical abnormalities in autistic rats treated with propionic acid. PPA treatment resulted in autistic behaviour in rats, including stereotyped repetitive behaviour, locomotion disturbances, anxiety, and decreased motor coordination and memory impairment. Furthermore, the current study’s neurochemical evaluation revealed enhanced oxidative stress, neuroinflammation, apoptosis, and neurotransmitter imbalance after ICV infusion of PPA in Wistar rats. These behavioural and neurochemical alterations are consistent with prior findings in PPA-treated autistic animal studies [31,54,78,79,80,81].

In this study, we developed a PPA-induced animal model of autism. Time-dependent behavioural changes in autistic rats and GST-treated rats at two distinct dosages were examined. Bodyweight assessments on several days revealed a considerable reduction in body weight in rats following 11 days of PPA injections. PPA, a short-chain fatty acid, has also been shown to cause weight loss in animals and humans [82], possibly due to increased gluconeogenesis or altered fatty acid metabolism [83,84].

This reduction in body weight increased in a dose-dependent manner after continuous GST administration, similar to the research report’s findings [42]. This investigation estimated the brain-body weight ratio by dividing the fresh brain weight by the bodyweight at the end of the protocol schedule. It was performed to understand the influence of PPA and GST on total brain mass in autistic conditions. The brain-body weight ratio declined considerably in PPA-induced experimental rats but increased dose-dependently following GST treatment.

During the forced swim test, the immobility period was used to assess the depression-like behavioural analysis that was evident in PPA-induced autistic rats. This study suggests an increase in immobility time following PPA administration on days 1, 13, 23 and 43, indicating an elevated depressive effect that was mitigated by prolonged GST treatment on the same days.

Likewise, the prior study found an enhanced depressed effect following PPA treatment [85]. According to previous findings, interleukin-6 elevates STAT3 levels and promotes depression by increasing serotonin transporters (SERT) [86,87]. The increased inflammatory cascade suggested by neurochemical analysis of brain homogenate and blood plasma could contribute to the increased inflammatory reactions. Several studies have found a link between depressive-like behaviour and inflammation caused by mitochondrial dysfunction and oxidative stress [88,89].

GST therapy mediates antidepressant benefits through activation of the cyclic AMP response element-binding protein (CREB) and brain-derived neurotrophic factor (BDNF) signalling pathways in a chronic unpredictable stress model of depression in mice [39]. These findings confirm our study’s hypothesis that GST reduced the depressive-like behaviour associated with autism.

Our actophotometer data showed decreased locomotion activity following PPA injection on day 1, 13, 23 and 43, which is consistent with the findings of previous investigations [54,57]. After GST treatment at 30 mg/kg and 60 mg/kg, there was a significant and dose-dependent improvement in locomotion behaviour on the same days, a primary hallmark of autistic patients. Numerous research findings showed decreased motor coordination in rodents following PPA injection [54,57,76,81].

BCT was used to assess muscle coordination by counting the number of slips that occurred while moving on the beam. Our findings reveal that PPA autistic rats had a higher frequency of slips on days 1, 13, 23 and 43, indicating poor neuromuscular coordination, which was decreased in a dose-dependent way by GST treatment on the same days.

We found that GST therapy restored muscular coordination in ethidium bromide-induced multiple sclerosis [42].

ASD individuals are generally recognized to have impaired spatial memory, cognitive impairments, and intellectual disability. The MWM is used to assess rodents’ spatial memory and cognitive abilities. The repeated ICV infusion of PPA was found in our experiment to impair spatial learning, as evidenced by increased ELT and a decrease in TSTQ on days 40, 41, 42, 43 and 44 [90].

These findings are comparable to indicate that memory is affected following multiple infusions of the PPA-treated ASD model [91]. When a high dose of GST was administered, there was a considerable drop in ELT and an increase in TSTQ, indicating a significant restoration of memory loss compared to a low dose on the same days.

The levels of STAT3 and PPAR gamma in both brain homogenate and CSF were measured to confirm the involvement of signalling processes in the pathology of autism. S3I-201 and flavonoids have previously been shown to target STAT3 in a model of autism, thereby improving the disease [14,92]. The level of STAT3 was found to be significantly higher in the brain homogenate and CSF of autistic rats treated with a PPA-induced experimental model of autism in adult rats. On the other hand, long GST treatment lowered STAT3 levels in a dose-dependent manner.

In addition, PPAR gamma was found to play an essential role in neuroinflammation by regulating the level of STAT3 [21].

Preclinical and clinical studies have shown selective PPAR gamma agonists to improve biochemical and behavioural changes associated with autism [31,93,94]. In our study, the ELISA level of PPAR gamma was increased following GST treatment in both rat brain homogenate and CSF, indicating an anti-inflammatory effect in PPA-treated rats.

The neurochemical evaluation results from our study provide strong evidence about GST’s neuroprotective function. Neurotransmitters, which play an important role in memory, mood, and behavioural regulation, must be balanced for appropriate neuronal development and functioning. One of the key causes of the emergence of behavioural traits in many neurological illnesses was neurotransmitter dysfunction. Several investigations have been undertaken to determine the significance of dysregulation of neurotransmission in the pathophysiology of autism by altering numerous development processes in the brain, such as cell differentiation and cell migration [95,96]. In autism, the most studied neurotransmitters include serotonin, dopamine, glutamate, and acetylcholine. Serotonin is linked to brain development, implying that it is altered in autistic conditions, and its deficit leads to socially impaired, repetitive, and depressive behaviour [97,98].

Along with a decrease in dopamine and Ach, an increase in glutamate was found to be responsible for autistic behaviour [99,100,101]. Increased glutamate levels activate microglia and cause neuroinflammation, whereas decreasing dopamine and acetylcholine levels modify neuronal excitability, resulting in mood changes. In accordance with previous findings, we observed a decrease in serotonin, dopamine, and acetylcholine and an increase in glutamate in PPA-induced autism in rats. This level was considerably recovered in a dose-dependent manner after long-term GST administration, leading to improved autistic behaviour.

The regulation and balance of the apoptotic cascade are required for cell survival, and disruption of this process can result in impaired neuronal growth, resulting in an autistic phenotype [102,103]. After PPA exposure, the levels of apoptotic markers such as Bax and Caspase 3 increased, but the anti-apoptotic marker Bcl-2 decreased [104]. Long-term GST administration has been shown to protect cells from apoptosis by reducing the levels of Bax and Caspase-3 and increasing the levels of Bcl-2.

According to various studies, increased oxidative stress caused by increased ROS generation and mitochondrial dysfunction may be a pathogenic feature of autism development [105,106,107].

In our investigation, the level of oxidative stress indicators was assessed in brain homogenates of PPA and GST-treated autistic rats to determine the severity of disease following PPA injection and the protective effect of GST against oxidative stress. Our findings show that PPA-induced autistic rats had higher levels of AchE, LDH, nitrite, and MDA, as well as lower levels of antioxidants, primarily SOD and GSH. Several investigations were undertaken to investigate the enhanced oxidative stress in brain homogenates of PPA-treated rats [68,81]. PPA-induced autistic rats treated with GST showed a significant decrease in oxidative stress markers, indicating its antioxidant effects, in a dose-dependent manner. GST’s antioxidant activities were also confirmed in a previous study on an ethidium bromide-induced multiple sclerosis model, supporting the above statement [42].

Clinical studies on autistic children clearly showed higher inflammatory cytokine levels in CSF, resulting in an immunological response and neuronal damage [108,109]. As previously stated, neuroinflammation is associated with depressive symptoms in ASD patients. It can also interfere with neural networks that control memory, decision-making ability, and behavioural features. In our investigation, PPA-treated autistic rats have higher levels of inflammatory markers such as TNF- α and IL-1β in brain homogenate and blood plasma, which is consistent with earlier findings [57]. After continuous GST administration, this enhanced inflammatory response was restored, protecting neurons from damage and inflammation. In autistic rats, high-dose GST treatment was more effective than low-dose GST treatment.

This study analysed the rat’s whole brain and coronal sections to study morphological alterations linked to autism. Chronic GST administration improved morphological alterations in PPA-treated autistic rats, such as reduced total brain mass, degenerated meninges, and constricted prefrontal cortex. Coronal sections of PPA-treated rats revealed deformed basal ganglia and a defragmented hippocampus area and degraded white matter.

Furthermore, an examination of demyelination volume in rat brains showed a significant decrease in white matter volume following PPA injections [68]. Compared to PPA-treated autistic rats, chronic therapy with GST30 mg/kg and GST60 mg/kg recovered the abnormalities in brain sections and enabled the remyelination of degraded areas. It was observed that continuous therapy with GST reduces gross pathogenic and morphological alterations.

Using advanced techniques, researchers discovered anomalies in the brain’s white matter associated with autism pathogenesis [110,111]. MBP expression was shown to be aberrant in samples from the cortex of ASD children in a post-mortem study. Nonetheless, no proposed mechanistic investigation indicates a direct link between MBP and autism [112]. The level of myelin basic protein in brain homogenate was found to be considerably lower following PPA injection in our investigation. The amount of myelin basic protein was significantly restored after long-term treatment with GST in a dose-dependent manner. Previous research has convincingly demonstrated the link of lower MBP protein levels in PPA-induced autistic rats [54].

Histological sections of rat brains from all groups revealed distinct alterations in the neuronal cells of the cerebral cortex. It has previously been observed that if the somatic size or shape of pyramidal neurons is considerably altered, their functional integrity is extremely likely to be disrupted [113]. This could be due to a decrease in dendritic complexity and axonal length, which has a negative impact on connection [114]. Autism neuropathology is characterized by a lack of stellate neurons, which results in microdysgenesis within the entorhinal cortex [115]. Oligodendrocytes were characterized by a tiny rounded or oval nucleus with a rich chromatin structure. Astrocytes had a circular or pale oval nucleus with condensed heterochromatin in granules [116,117]. Microglial cells were distinguished by their connection with capillaries, rod shape, and elongated nuclei [118]. Oligodendrocytes are preferentially affected in the brains of autistic patients [119] and give rise to behavioural deficits, as reported in preclinical studies [120]. Morphological changes in astrocytes like membranous blebs and the presence of reactive microglia are prominently observed in ASD brains [121]. According to our histological findings, the healthy structure of the cerebral cortex neurons is altered in the propionic acid-induced animal model of autism. Neuronal cell degeneration, including pyramidal cells, stellate cells, oligodendrocytes, and astrocytes, was found in the propionic acid-treated group, along with the appearance of rod-shaped microglial cells. As indicated in the propionic acid-treated group, the disturbed, irregular, and oval-shaped pyramidal cell layer is responsible for poor connectivity among cortical regions, especially those involved in social processing in autistic conditions. In our investigation, the propionic acid-treated group also had an altered shape of stellate neurons, consistent with earlier research. Our histological study revealed a loss of oligodendrocytes, the appearance of irregular-shaped astrocytes, and the presence of rod-shaped microglial cells in the propionic acid-treated group.

Our current research focuses solely on the neuroprotective potential of GST by altering the JAK-STAT/PPAR gamma signalling pathway and thereby alleviating behavioural, neurochemical, morphological, gross pathological, and histological changes in the PPA-induced experimental model of autism in adult rats. This study suggests that all of the neurochemical characteristics investigated could be employed as a viable biomarker for disease detection in the early stages. As a result, it is essential to conduct a mechanistic investigation to confirm the role of signalling pathways in the progression of autism. Molecular information found through western blotting could potentially be used to support the hypothesis. A gender-based study could also be conducted to investigate the gender association in autism.

## 5. Conclusions

In conclusion, this study strongly suggested that GST had neuroprotective effects in autistic rats treated with propionic acid. It also proved GST’s anti-oxidant and anti-apoptotic potential and anti-inflammatory properties by altering the JAK-STAT and PPAR-gamma signalling pathway. Based on our histological findings, we may conclude that the degeneration of numerous neuronal cells in the cerebral cortex region of the rat brain determines the significant neuropathological abnormalities in autism. Propionic acid-treated brains could show autistic neuronal defects. The regeneration and repair of neuronal cells displayed in histological sections demonstrate GST’s effectiveness.

In summary, these findings point to GST as a promising compound that could be employed to treat autism in the future. It also emphasizes the importance of future studies based on mechanistic and confirmatory molecular mechanisms, including STAT3 and PPAR-gamma knock-in and knock-out mice. Aside from that, we can state that assessing STAT3 and PPAR-gamma levels can be a viable biomarker for autism diagnosis due to their participation in disease development and severity.

## 6. Chemical Compounds Studied in This Article

**Guggulsterone** (PubChem CID: 6450278) https://pubchem.ncbi.nlm.nih.gov/compound/Guggulsterone (accessed on 24 August 2020).

**Propionic acid** (PubChem CID: 1032) https://pubchem.ncbi.nlm.nih.gov/compound/Propionic-acid (accessed on 24 August 2020).

## Figures and Tables

**Table 1:** Neuroprotective potential of guggulsterone on STAT3, PPAR-gamma, and myelin basic protein level in Propionic acid-induced experimental model of autism in adult rats.

S. no.	Groups	STAT3		PPAR-Gamma		Myelin Basic Protein
		Brain Homogenate (nM/µg Protein)	CSF (ng/mL)	Brain Homogenate (nM/µg Protein)	CSF (ng/mL)	Brain Homogenate (µg/mg Protein)
1.	Vehicle Control	1.72 ± 0.038	0.29 ± 0.004	6.173 ± 0.041	2.05 ± 0.042	106.3 ± 0.325
2.	Sham Control	1.73 ± 0.052	0.29 ± 0.006	6.293 ± 0.036	2.06 ± 0.049	106.2 ± 0.422
3.	GST60 Perse	1.76 ± 0.050	0.31 ± 0.005	6.397 ± 0.069	2.10 ± 0.051	107.1 ± 0.146
4.	PPA	6.33 ± 0.057 *	0.69 ± 0.007 *	2.388 ± 0.109 *	0.83 ± 0.030 *	61.28 ± 0.313 *
5.	PPA + GST30	4.83 ± 0.045 ^@^	0.59 ± 0.003 ^@^	3.333 ± 0.037 ^@^	1.35 ± 0.022 ^@^	77.44 ± 0.252 ^@^
6.	PPA + GST60	3.18 ± 0.029 ^@#^	0.50 ± 0.003 ^@#^	4.482 ± 0.088 ^@#^	1.73 ± 0.021 ^@#^	92.55 ± 0.157 ^@#^

Statistical analysis followed by one-way ANOVA (post hoc Tukey’s test). Values expressed as mean ± SEM (*n* = 6 rats per group). * *p* < 0.001 v/s vehicle control, sham control and GST60 perse; @ *p* < 0.001 v/s PPA; @# *p* < 0.001 v/s PPA + GST30.

**Table 2:** Neuroprotective potential of guggulsterone on apoptotic markers in propionic acid-induced experimental model of autism in adult rats.

S. no.	Groups	Caspase-3		Bax		Bcl-2	
		Brain Homogenate (nM/mg Protein)	Blood Plasma (ng/mL)	Brain Homogenate (ng/mg Protein)	Blood Plasma (ng/mL)	Brain Homogenate (ng/mg Protein)	Blood Plasma (ng/mL)
1.	Vehicle Control	106.10 ± 1.103	1.94 ± 0.014	5.35 ± 0.069	1.06 ± 0.013	30.23 ± 0.366	8.84 ± 0.016
2.	Sham Control	105.30 ± 0.454	1.94 ± 0.014	5.23 ± 0.056	1.05 ± 0.014	30.53 ± 0.359	8.83 ± 0.012
3.	GST60 Perse	105.70 ± 0.355	1.95 ± 0.011	5.31 ± 0.074	1.06 ± 0.014	29.86 ± 0.501	8.83 ± 0.011
4.	PPA	159.90 ± 0.549 *	6.73 ± 0.022 *	12.14 ± 0.308 *	5.83 ± 0.012 *	20.24 ± 0.299 *	2.58 ± 0.009 *
5.	PPA + GST30	145.92 ± 0.436 ^@^	4.66 ± 0.101 ^@^	9.54 ± 0.126 ^@^	4.46 ± 0.016 ^@^	24.13 ± 0.256 ^@^	4.66 ± 0.029 ^@^
6.	PPA + GST60	134.60 ± 1.073 ^@#^	3.68 ± 0.033 ^@#^	7.31 ± 0.118 ^@#^	3.27 ± 0.025 ^@#^	26.60 ± 0.168 ^@#^	6.97 ± 0.017 ^@#^

Statistical analysis followed by one-way ANOVA (post hoc Tukey’s test). Values expressed as mean ± SEM (*n* = 6 rats per group). * *p* < 0.001 v/s vehicle control, sham control and GST60 perse; @ *p* < 0.001 v/s PPA; @# *p* < 0.001 v/s PPA + GST30.

**Table 3:** Neuroprotective potential of guggulsterone on neurotransmitters level in propionic acid-induced experimental model of autism in adult rats.

S. no.	Groups	Serotonin (ng/mg Protein)	Glutamate (ng/mg Protein)	Dopamine (ng/mg Protein)	Ach (ng/mg Protein)
1.	Vehicle Control	39.15 ± 0.542	111.80 ± 0.400	90.84 ± 0.566	11.62 ± 0.177
2.	Sham Control	40.84 ± 0.478	111.60 ± 0.409	90.93 ± 0.594	11.68 ± 0.107
3.	GST60 Perse	39.55 ± 0.607	111.50 ± 0.549	91.07 ± 0.475	11.79 ± 0.181
4.	PPA	14.12 ± 0.133 *	308.60 ± 2.029 *	31.70 ± 0.527 *	5.81 ± 0.127 *
5.	PPA + GST30	17.17 ± 0.256 ^@^	214.50 ± 2.314 ^@^	49.23 ± 0.335 ^@^	7.28 ± 0.102 ^@^
6.	PPA + GST60	24.65 ± 0.373 ^@#^	184.20 ± 1.937 ^@#^	54.12 ± 1.063 ^@#^	9.46 ± 0.125 ^@#^

Statistical analysis followed by one-way ANOVA (post hoc Tukey’s test). Values expressed as mean ± SEM (*n* = 6 rats per group). * *p* < 0.001 v/s vehicle control, sham control and GST60 perse; @ *p* < 0.001 v/s PPA; @# *p* < 0.001 v/s PPA + GST30.

**Table 4:** Neuroprotective potential of guggulsterone on inflammatory cytokines in propionic acid-induced experimental model of autism in adult rats.

S. no.	Groups	TNF-α		IL-1β	
		Brain Homogenate (pg/mg protein)	Blood Plasma (pg/mL)	Brain Homogenate (pg/mg Protein)	Blood Plasma (pg/mL)
1.	Vehicle Control	31.04 ± 0.379	30.73 ± 0.440	13.60 ± 0.223	16.58 ± 0.147
2.	Sham Control	31.33 ± 0.430	30.82 ± 0.481	13.48 ± 0.175	16.33 ± 0.143
3.	GST60 Perse	30.69 ± 0.368	31.69 ± 0.305	14.02 ± 0.274	16.66 ± 0.108
4.	PPA	71.59 ± 0.371 *	102.00 ± 0.389 *	24.51 ± 0.207 *	92.29 ± 0.120 *
5.	PPA + GST30	62.34 ± 0.132 ^@^	81.54 ± 0.397 ^@^	20.32 ± 0.364 ^@^	76.47 ± 0.110 ^@^
6.	PPA + GST60	51.34 ± 0.610 ^@#^	55.47 ± 0.378 ^@#^	18.41 ± 0.097 ^@#^	52.46 ± 0.159 ^@#^

Statistical analysis followed by one-way ANOVA (post hoc Tukey’s test). Values expressed as mean ± SEM (*n* = 6 rats per group). * *p* < 0.001 v/s vehicle control, sham control and GST60 perse; @ *p* < 0.001 v/s PPA; @# *p* < 0.001 v/s PPA + GST30.

**Table 5:** Neuroprotective potential of guggulsterone on oxidative stress in propionic acid-induced experimental model of autism in adult rats.

S.no.	Groups	AchE μM/mg protein)	LDH (Unit/mg protein)	SOD (µM/mg protein)	GSH (µM/mg protein)	Nitrite (µM/mg protein)	MDA (nM/mg protein)
1.	Vehicle Control	17.74 ± 0.215	111.20 ± 0.520	469.30 ± 0.756	32.43 ± 0.189	7.07 ± 0.034	31.24 ± 0.964
2.	Sham Control	18.42 ± 0.277	111.50 ± 1.050	468.80 ± 0.441	32.65 ± 0.326	7.05 ± 0.061	30.82 ± 0.476
3.	GST60 Perse	17.67 ± 0.214	112.1 ± 1.120	470.70 ± 1.602	32.13 ± 0.402	7.08 ± 0.041	31.44 ± 0.500
4.	PPA	54.11 ± 0.419 *	401.20 ± 2.100 *	320.90 ± 0.540 *	11.42 ± 0.153 *	9.03 ± 0.088 *	72.07 ± 0.869 *
5.	PPA + GST30	38.83 ± 0.514 ^@^	307.30 ± 4.054 ^@^	366.10 ± 0.621 ^@^	18.40 ± 0.171 ^@^	8.11 ± 0.045 ^@^	65.74 ± 0.526 ^@^
6.	PPA + GST60	31.18 ± 0.357 ^@#^	282.40 ± 3.630 ^@#^	384.70 ± 2.096 ^@#^	23.08 ± 0.167 ^@#^	7.55 ± 0.058 ^@#^	56.52 ± 0.832 ^@#^

Statistical analysis followed by one-way ANOVA (post hoc Tukey’s test). Values expressed as mean ± SEM (*n* = 6 rats per group).* *p* < 0.001 v/s vehicle control, sham control and GST60 perse; @ *p* < 0.001 v/s PPA; @# *p* < 0.001 v/s PPA + GST30.

## Data Availability

All data generated or analysed during this study are included in this article. There are no separate or additional files.

## Sample Availability

Samples of the compounds are not available from the authors.

## Abbreviations

15d-PGJ2	15-deoxy-Δ12,14-prostaglandin J2
Ach	Acetylcholine
AchE	Acetylcholinesterase
AP	Anterior/posterior
ASD	Autism spectrum disorder
BCT	Beam crossing task
BNDF	Brain-derived neurotrophic factor
BTBR	BTBR T+tf/J
CREB	Cyclic AMP response element binding protein
CSF	Cerebrospinal fluid
DV	Dorsal/ventral
ELT	Escape latency time
FST	Forced swim test
FXR	FarnesoidX receptor
GST	Guggulsterone
IAEC	Institutional Animal Ethics Committee
ICV-PPA	Intracerebroventricular-propionic acid
IL-1β	Interleukin 1 beta
JAK	Janus kinase
LDH	Lactate dehydrogenase
MBP	Myelin basic protein
MDA	Malondialdehyde
ML	Medial/lateral
MWM	Morris water maze
NO	Nitric oxide
PPAR	Peroxisome proliferator-activated receptor
SHP-1	Srchomology 2 domain-containing protein tyrosine
SOD	Superoxide dismutase
STAT3	Signal transducer and activator of transcription 3
TNF-α	Tumornecrotic factor-alpha
TSTQ	Time spent in target quadrant
